# Soil texture mediates microbial assembly and network organization under moisture stress

**DOI:** 10.21203/rs.3.rs-10233190/v1

**Published:** 2026-08-03

**Authors:** Muhammad Shoib Nawaz, Muhammad Suleman, Vidya Suseela, Barbara J. Campbell

**Affiliations:** 1 Department of Biological Science, Clemson University, SC, USA; 2 Department of Plant & Environmental Sciences, Clemson University, Clemson, SC, USA

**Keywords:** Rhizosphere microbiome, Soil texture, Soil moisture, Co-occurrence Networks, Microbial community assembly, *Andropogon virginicus*

## Abstract

**Background:**

Climate-driven changes in moisture are expected to intensify environmental selection in soils, yet the extent to which soil texture modifies microbial responses to moisture stress remains poorly understood. Because soil texture governs water retention, nutrient diffusion, and habitat structure, it may fundamentally influence how microbial communities assemble and function under drought. Here, we investigated how moisture and soil texture interact to shape bacterial and fungal rhizosphere communities associated with *Andropogon virginicus*, a drought-tolerant wild relative of maize, across 28 natural field sites in the southeastern United States.

**Results:**

Soil texture explained greater variation in microbial community composition than moisture stress alone, while moisture-texture interactions produced distinct community structures across sites. Distance-based redundancy (dbRDA) analyses identified soil nitrogen pools, pH, organic matter, and micronutrients as major environmental drivers. Community assembly analyses showed that xeric conditions strengthened deterministic assembly in bacterial communities through increased homogeneous selection, whereas fungal communities remained predominantly stochastic across textures. Moisture-texture interactions also restructured microbial co-occurrence networks, with bacterial networks under xeric conditions exhibiting greater connectivity, competitive interactions, and phylogenetic clustering, while fungal networks remained comparatively modular and texture dependent. Predicted functional profiles further revealed texture-dependent shifts in bacterial metabolic pathways and fungal ecological guilds across moisture regimes, indicating coordinated changes in microbial functional organization under drought.

**Conclusions:**

Our findings demonstrate that soil texture mediates microbial responses to moisture stress by modifying community assembly, ecological interactions, and functional organization rather than simply altering microbial diversity. Bacterial communities exhibited stronger deterministic responses to drought than fungal communities, highlighting contrasting ecological strategies across microbial domains. These results emphasize the importance of integrating soil physical properties with moisture stress when investigating rhizosphere microbiomes and provide ecological insights that may inform microbiome-based strategies for improving crop resilience under increasingly frequent drought conditions.

## 1 INTRODUCTION

Climate change is one of the important challenges of the 21st century, with far-reaching consequences for agricultural productivity and ecosystem services. Among many environmental factors associated with climate change, warming and precipitation shifts are driving earlier and more severe drought events worldwide [1, 2]. This pattern is evident in the southeastern US, where increased temperatures and uneven rainfall distribution have resulted in drought events [3]. Drought directly affects the physiology of plants, the physicochemical properties of soil and the structure and functions of microbial communities. Besides, the direct effects of drought on microbial communities, stressed plants, and soils may also indirectly influence them [4]. Historically, these soil communities serve as major allies of plants to facilitate the acquisition of water and nutrients and alleviate a(biotic) stresses [5]. They are highly sensitive to fluctuations in soil conditions, such as temperature, nutrient and water content. Soil water content is important for microbes because dry soil slows microbial metabolism by disrupting the osmoregulation and reducing nutrient diffusion [6, 7]. Moreover, soil moisture levels indirectly impact microbial composition and function by changing soil redox potential, ionic strength and pH. Ultimately, changes in microbial communities affect plant and soil health, and vice versa, leading to a feedback cycle [8].

Drought conditions provide an opportunity to understand how drought-adapted plant species shape microbial communities within diverse environments. *Andropogon virginicus* L. (broomsedge bluestem) can be a valuable model plant in this regard. It is a native perennial C4 grass widely distributed across the southeastern US and well known for its tolerance to poor soils, fire, and extended dry periods. It grows naturally, and forms dense monoculture stands in abandoned agricultural fields, along roadsides, and on disturbed and poorly managed lands [9]. The evolutionary proximity of *A. virginicus* to agronomically important cereal crops such as maize, sorghum, and millet makes it even more compelling as a model system [10]. This close phylogenetic relationship raises the possibility that characterizing its associated microbiome could inform strategies to improve stress tolerance in modern crop systems. Recent studies have shown that inoculating maize with the rhizobiome of *A. virginicus* significantly enhances drought tolerance by improving growth, photosynthetic activity, and oxidative stress tolerance under water-limited conditions [11, 12]. A metagenomic study of the related species, *A. gerardii*, rhizobiome identified a *Pseudomonas* sp. recovered as a metagenome-assembled genome (MAG) with genes related to drought stress response [13]. Cultivating and inoculating *Pseudomonas* sp. confirmed its ability to alleviate drought stress in the host plant. Despite its resilient nature, the microbiome of *A. virginicus* remains understudied under natural environments where soil water content, texture, and geographic locations vary, except for one study that examined its leaf fungal community and mycotoxins across different sites, finding that geography and visible black-stroma fungal structures shaped fungal community composition [14].

Soil chemical parameters, such as nutrients and pH, are significant determinants of microbial diversity; for instance, acidic soils support the growth of microbes adapted to low pH [15]. Macronutrients such as nitrogen (N) and phosphorus (P) serve as essential substrates for microbial metabolism, potassium (K) and calcium (Ca) play regulatory and structural roles, and trace elements such as copper, zinc, manganese, and boron either function as enzyme cofactors or exert toxicity at higher concentrations [8]. Organic matter provides energy for microbial cells, but is often less available under drought due to lower plant biomass and exudation, leading to a shift in microbial communities toward stress-tolerant and resource-efficient taxa [5]. Drought significantly alters soil physicochemical properties, which affect both plants and microbes [16, 17]. Reduced water availability lowers soil and aggregate porosity, thereby limiting the mobility of nutrients such as P, K, Ca, and trace elements such as copper, manganese, and zinc [18]. These changes limit microbial survival, competition, and activity, making water a key ecological filter. Moreover, different soil textures, such as sandy, loamy, and clayey soils, respond differently to drought stress due to their differences in water-holding capacity and microhabitat structure. Texture can further add to the effects of drought on soil, plants, and microbes [19]. Loamy soils contain an appropriate ratio of silt, sand, and clay particles to hold water better than sandy soils, which, in contrast, rapidly lose water through their large pores [20]. Therefore, loamy soils support slow-growing microbial taxa, but sandy soils favor fast-growing taxa under drought [21]. Soil texture also influences plant root growth, penetration, and exudation patterns, thereby shaping rhizosphere microbiomes [22]. Altogether, interactions among soil texture, moisture, pH, nutrients, and redox gradients generate a heterogeneous matrix of ecological niches that shape microbial community composition, activity, and resilience under drought stress [23].

The present study investigates how moisture and soil texture interact to shape the rhizobiome of *A. virginicus* across 28 natural field sites. To understand the ecological processes governing microbial assembly in this drought-tolerant ruderal grass, we examined microbial diversity, community composition, assembly processes, co-occurrence network organization, and predicted functional potential across mesic and xeric conditions within contrasting soil textures. We tested two primary hypotheses. First, we hypothesized that interactions between soil moisture and texture are major determinants of rhizosphere microbiome structure and assembly in *A. virginicus*. Second, we hypothesized that bacterial and fungal communities exhibit distinct ecological responses to moisture–texture gradients and rhizosphere selection. Together, this framework enables us to evaluate how interacting physical and chemical soil properties influence microbial assembly, ecological interactions, and functional potential under natural moisture gradients.

## 2 MATERIALS AND METHODS

### 2.1 Sample Collection

During the late summers of 2023 and 2024, soil samples were collected from multiple sites across the southeastern United States, targeting naturally occurring populations of *Andropogon virginicus* L. (broomsedge bluestem), a native grass species known for its drought and abiotic stress tolerance. Sampling locations were identified via the SouthEast Regional Network of Expertise and Collections (SERNEC) data portal (https://sernecportal.org) [24] and iDigBio (https://www.idigbio.org/) [25]. Sites were selected based on GPS coordinates and the presence of at least 10 m^2^ of continuous *A. virginicus* ground cover. All necessary permissions were obtained prior to sampling. Detailed site information, including coordinates and descriptions, is provided in Supplementary S1.

At each site, rhizosphere and bulk soil samples were collected following a modified protocol from McPherson et al. [26]. Plants were carefully uprooted with minimal disturbance to roots. Rhizosphere soil was collected by gently shaking roots to dislodge loosely attached particles; the remaining soil was transferred into sterile Whirl-Pak^®^ bags. A 0.25 g subsample of rhizosphere soil was immediately preserved in DNA/RNA Shield (Zymo Research) for downstream molecular analysis. Bulk soil was collected from the same depth, away from the root zone. Five replicate samples were taken per site. All samples were stored on dry ice in the field and transferred to −80 °C upon arrival at the lab. Additional soil was collected and stored separately on ice for physicochemical analysis, then later at 4 °C.

### 2.2 Soil physicochemical properties

All soil samples were submitted to the Clemson University Agricultural Service Laboratory for analysis, including pH and nutrient concentrations (e.g., P, K, Ca, Mg, and micronutrients). Soil texture was assessed using the 2-hour hydrometer method [27]. Briefly, air-dried soil was sieved to <2 mm, weighed, and treated with 0.5 % sodium hexametaphosphate for dispersion. After mechanical mixing, the suspension was transferred into 1000 mL sedimentation cylinders. Hydrometer readings were taken at 40 seconds and 2 hours to calculate sand, silt, and clay proportions based on sedimentation rates according to Stokes’ Law. Temperature corrections and blank controls were applied. Textural classes were assigned using the USDA soil texture triangle (https://www.nrcs.usda.gov/resources/education-and-teaching-materials/soil-texture-calculator). Soil moisture content was determined using fresh and oven-dried soil weights. Subsamples were weighed immediately after collection (fresh weight), dried at 105 °C for 72 h to constant mass, and reweighed to obtain dry weight. Moisture content was calculated as the difference between fresh and dry weight divided by dry weight and expressed as a percentage. Sites were classified into xeric and mesic groups based on soil moisture content (Supplementary Table S2), with xeric soils characterized by consistently low moisture content and mesic soils by substantially higher moisture levels (typically <5% and >10%, respectively). These groupings were used as the StressType variable in all downstream analyses.

### 2.3 DNA extraction and sequencing

Total genomic DNA was extracted using the Quick-DNA Fecal/Soil Microbe 96 Kit (Zymo Research) following the manufacturer’s instructions. DNA was quantified and normalized to 1 ng/μL prior to PCR. For bacterial community profiling, the V3-V4 region of the 16S rRNA gene was amplified using primers 341F and modified 806R with dual indexed barcodes and Illumina overhang adapters [28–30] and Q5^®^ High-Fidelity DNA Polymerase (New England Biolabs). PCR cycling conditions for the 16S rRNA gene region included an initial denaturation at 98 °C for 30 s, followed by 35 cycles of 98 °C for 10 s, 55°C for 30 s, and 72 °C for 60 s, with a final extension at 72 °C for 2 min. Successful amplification was confirmed by gel electrophoresis, with target bands observed between 400-500 bp. Similarly, for fungal community analysis, the ITS2 region was amplified using the ITS3 and ITS4 primers [31], modified with Illumina overhang adapters as described in the dual-index sequencing strategy [28] and using the same enzyme. The ITS2 PCR conditions include an initial denaturation at 98 °C for 45 s, followed by 38 cycles of 98 °C for 10 s, 55 °C for 30 s, and 72 °C for 60 s, and a final extension at 72 °C for 2 min. Expected amplicons (~400 bp) were confirmed by electrophoresis. PCR products were purified using sbeadex^™^ SAB magnetic beads (LGC, Biosearch Technologies) and quantified via Qubit^™^ dsDNA High Sensitivity Assay (Thermo Fisher). Final libraries were cleaned, normalized, and pooled equimolarly. Library quality and molarity were assessed on Agilent TapeStation 4200. Sequencing was conducted on an Illumina NextSeq platform using paired-end reads.

### 2.4 Amplicon Sequence Analysis

#### 2.4.1 QIIME2

Raw paired-end reads were imported into QIIME 2 using a manifest-based format [32]. Quality control and demultiplexing summaries were generated using the demux summarize plugin. DADA2 was used for denoising, chimera removal, and ASV generation with trimming parameters optimized per dataset [33]. Forward and reverse reads were trimmed to retain high-quality regions. Low-abundance ASVs (<10 reads) were removed. Taxonomic classification for 16S rRNA gene was done via classify-sklearn using a pre-trained Naive Bayes classifier with the SILVA 138 99% database [34]. For ITS2, classification used the UNITE v8 99% dynamic classifier [35]. Non-target features, including chloroplast, mitochondrial, eukaryotic, and unassigned sequences, were filtered from the 16S rRNA gene data feature table. Only fungal sequences were retained for ITS data. A phylogenetic tree was constructed using MAFFT alignment, masking [36], FastTree inference, and midpoint rooting [37]. All key QIIME 2 artifacts (feature tables, representative sequences, taxonomy, and trees) were exported for downstream analyses in R [32].

#### 2.4.2 Microbial Community Analysis

Microbial community analysis was performed in R using phyloseq [38] and microeco [39]. ASV tables, taxonomy annotations, metadata, and phylogenetic trees were assembled into a phyloseq object. Non-prokaryotic taxa (for 16S rRNA gene) and non-fungal sequences (for ITS) were removed based on taxonomic assignment in the ASV table, excluding ASVs classified as Eukaryota for 16S rRNA gene datasets (and retaining only taxa assigned to the kingdom Fungi for ITS datasets. ASVs with fewer than 10 total counts were excluded. Samples with insufficient depth ≤10 total counts were filtered, and data were rarefied at fixed sequencing depths (16S rRNA gene: 15,444 reads; ITS: 10,660 reads) to standardize sampling effort and were used only for analyses requiring equal library sizes, such as alpha diversity. The curated phyloseq objects were then converted to microeco microtable format to enable downstream ecological analyses. Relative abundances were computed at multiple taxonomic levels, and class-level relative abundances were visualized for each Texture and StressType combination using the trans_abund module with the top 20 classes plotted using the ggnested package [39].

##### 2.4.2.1 Alpha and Beta Diversity Analysis

Alpha diversity analyses were conducted on rarefied datasets. Multiple alpha diversity metrics, including Observed richness, Shannon diversity, Berger-Parker dominance, Pielou’s evenness and and Faith’s phylogenetic diversity (PD) were computed using *microeco*, and group differences were evaluated with non-parametric Kruskal-Wallis (KW) tests and Dunn’s post hoc comparisons [39]. Diversity responses were visualized using violin-boxplot overlays across StressType, Texture, and StressTexture, including a faceted visualization of StressType effects within each Texture.

Beta diversity was assessed using Weighted UniFrac distances computed in *microeco* [40]. PCoA ordinations were visualized with confidence ellipses grouped by StressType, Texture, and StressTexture, including a faceted visualization of StressType and SoilType effects within each Texture. Group differences were tested using PERMANOVA [41] and PERMDISP (dispersion homogeneity) [42].

##### 2.4.2.2 Differential Abundance Testing

For both 16S rRNA gene and ITS datasets, differential abundance across StressType groups was evaluated using LEfSe (KW test followed by Wilcoxon pairwise tests, linear discriminant analysis (LDA) > 2.0) [43] and ANCOM-BC2 was used with bias correction and Benjamini-Hochberg (BH) false discovery rate (FDR) adjustment; taxa with q ≤ 0.05 were considered significant [44]. Only consensus significant genera detected by both methods were retained. From these, we visualized the top 10 mesic-enriched and top 10 xeric-enriched genera based on LEfSe LDA scores, and the top 10 positive (mesic-enriched) and top 10 negative (xeric-enriched) ANCOM-BC2 Log fold changes (LFCs).

For StressTexture comparisons, differential abundance was assessed using KW tests with Dunn’s post-hoc comparisons (BH-corrected p ≤ 0.05) and ALDEx2 (CLR transformation with effect size > |1| and BH-adjusted p ≤ 0.05) [45, 46]. Consensus significant genera shared between KW-Dunn and ALDEx2 were extracted, and the top 10 most abundant responding taxa were plotted separately for 16S rRNA gene and ITS.

##### 2.4.2.3 Distance-based redundancy (dbRDA) analysis of microbial communities and environmental drivers

dbRDA was used to evaluate relationships between microbial community composition and environmental variables using weighted UniFrac distance matrices. Analyses were conducted in R using the vegan package [40], with constrained ordination performed via capscale [47, 48]. Prior to modeling, environmental variables were screened for multicollinearity using variance inflation factors (VIF), and variables with VIF > 10 were iteratively removed to obtain a reduced set of independent predictors [49]. The final model included soil pH, organic matter, and macro- and micronutrients, along with StressType (mesic vs. xeric) and soil texture, as categorical predictors, and their interaction. Model significance was assessed using permutation-based ANOVA (999 permutations) and environmental vectors were fitted to ordination space using envfit to identify variables significantly correlated with community structure (p < 0.05), and only significant predictors were retained for visualization.

##### 2.4.2.4 Community assembly inference

Community assembly processes were quantified using a null model framework integrating β-nearest taxon index (βNTI) and a Raup-Crick metric based on Bray-Curtis dissimilarity (RCbray) [50, 51]. Phylogenetic turnover was estimated as β-mean nearest taxon distance (βMNTD), and βNTI values were calculated by comparing observed βMNTD to null distributions generated by randomizing phylogenetic relationships among taxa (450 permutations). βNTI values > +2 and < −2 were interpreted as variable selection and homogeneous selection, respectively. For comparisons where |βNTI| ≤ 2, RCbray was used to further resolve non-selection processes by comparing observed Bray-Curtis dissimilarities to null expectations generated from randomized community abundance matrices (499 permutations). RCbray values > 0.95 and < −0.95 indicated dispersal limitation and homogenizing dispersal, respectively, whereas intermediate values indicated drift. This hierarchical framework partitions community assembly into deterministic and stochastic ecological processes.

##### 2.4.2.5 Co-occurrence Network Analysis

Co-occurrence networks for 16S rRNA gene and ITS datasets were inferred separately using the *microeco* package [39]. For each dataset, ASVs were retained only if present in ≥50% of samples, and abundance matrices were Hellinger-transformed [52] prior to correlation analysis. Networks were constructed using SPARCC (filter_thres = 0.0005; |r| ≥ 0.2; pseudo-p ≤ 0.05; FDR correction using the Benjamini–Hochberg method; 1,000 bootstrap iterations) because SPARCC is specifically designed for compositional microbiome data and reduces spurious correlations arising from relative abundance data [53]. Node tables included ASV-level taxa annotated as Phylum, Family, Genus, and degree; edge tables retained sign (+/−) and absolute weight. Network topology (node number, edge number, mean degree, clustering coefficient, density, and modularity) was quantified, and modules were detected using the Walktrap algorithm [54]. Node and edge overlap across StressType and StressTexture networks were assessed. Phylogenetic distance matrices were computed, and group differences in distances were evaluated using KW tests, followed by Dunn’s test [55, 56] with BH-adjusted p-values and compact letter display (CLD) assignment (α = 0.05). Standardized effect sizes (SES_MPD, SES_MNTD) were computed using 999 random-taxon null permutations, maintaining observed richness [39, 57].

Network robustness was evaluated using iterative perturbation simulations under three attack scenarios: (i) random edge removal, (ii) strong-edge removal, and (iii) hub-node removal across removal ratios of 0-0.99 [58]. Global efficiency (Eff) was used as the primary stability metric, and robustness was summarized as the area under the efficiency curve (AUC) for each network [59]. Node-level vulnerability indices were also computed. Network visualizations were produced using tidygraph [60] and ggraph [61], displaying nodes scaled by degree and fill colored by Phylum and node edge colored by module, and edges colored by interaction sign. The significance of modularity for each network was tested using degree-preserving null models (499 rewired networks), and p-values were calculated as *P*(Q_null ≥ Q_obs) [62].

##### 2.4.2.6 Functional Profile Prediction

MetaCyc pathway abundances were inferred from 16S rRNA gene sequences using PICRUSt2 with EC-stratified metagenome reconstruction [63]. Prediction confidence was evaluated using weighted nearest sequenced taxon index (NSTI) scores. Pathway abundance tables were converted to percent relative abundance per sample and aligned with sample metadata prior to downstream analysis. Predicted functional differences within each texture were compared between the mesic and xeric treatments at the individual pathway level using KW tests with BH-FDR correction (FDR < 0.05). For each pathway, group means and medians were calculated. Enrichment direction was determined from relative mean differences, and effect sizes were estimated using Cliff’s delta. Significant pathways were annotated using MetaCyc reference mappings [64] and filtered to retain those meeting statistical significance (FDR < 0.05) for downstream visualization and interpretation.

Fungal ecological guilds were assigned from ITS amplicon data using the FUNGuild database [65] within the microeco framework via the trans_func module [39] which generates both functional annotations and abundance-weighted functional redundancy (FR). Functional profiles were calculated as abundance-weighted guild contributions per sample and normalized to relative abundance. Guild composition was summarized at the StressTexture level using group means for visualization. Statistical differences in guild structure were assessed within each texture using Wilcoxon rank-sum tests (mesic vs. xeric), with BH-FDR correction (FDR < 0.05). Effect size and direction were calculated as differences in mean relative abundance between StressType groups, and enrichment was assigned based on the sign of the difference. Guilds were retained for visualization and interpretation based on statistical significance (FDR < 0.05) and/or a minimum effect size threshold (|Δ abundance| > 0.003).

## 3 Results

### 3.1 Soil physicochemical gradients vary across moisture stress and texture classes

**Figure 1.**
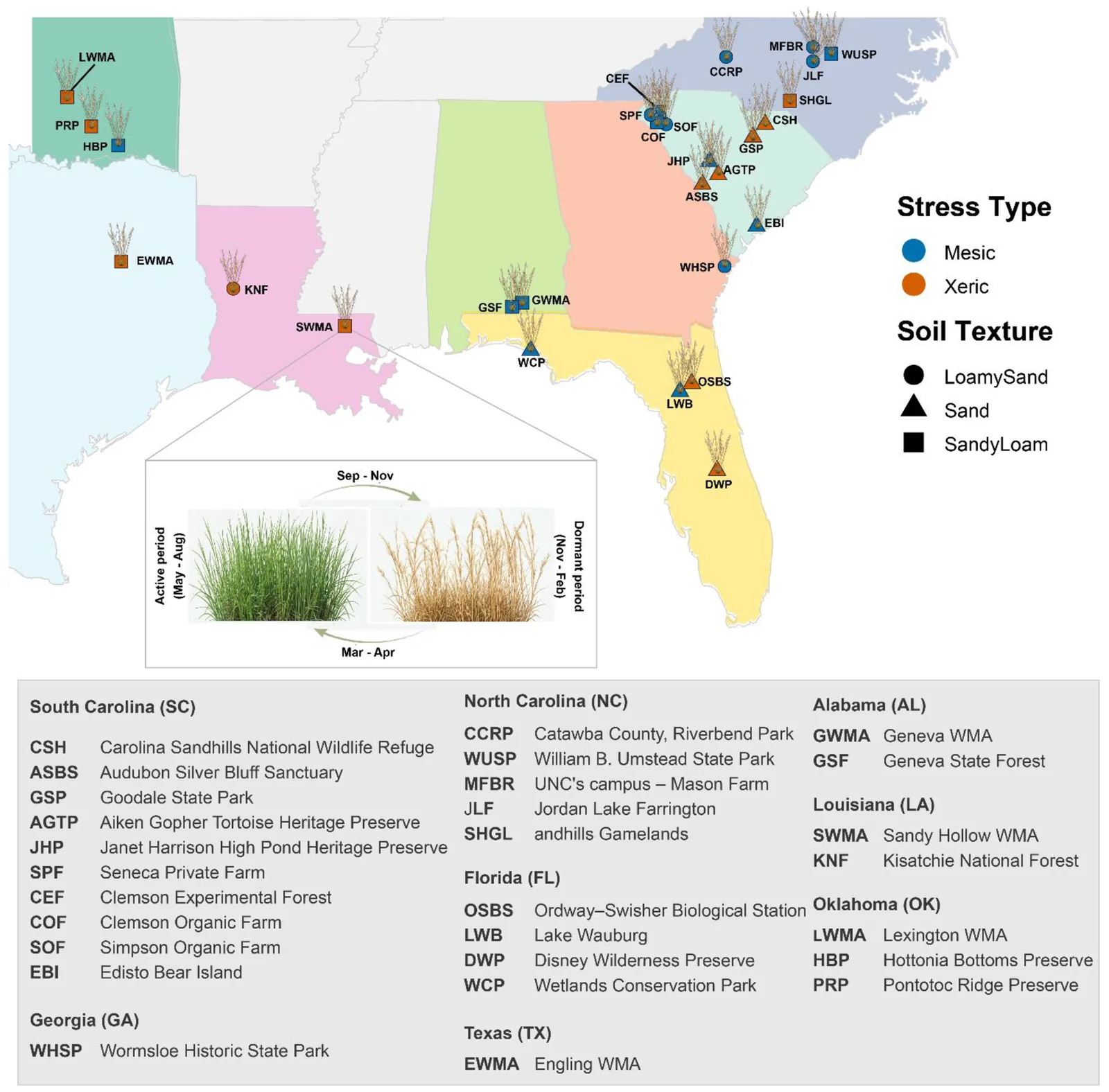
Sampling design across moisture and soil texture gradients. Map of sampling sites across the southeastern United States. Sites are classified by stress type (Mesic, blue; Xeric, orange) and soil texture (LoamySand, circles; Sand, triangles; SandyLoam, squares). Site codes correspond to locations listed below. The inset shows the seasonal growth cycle of *Andropogon virginicus.*

Sampling sites spanned multiple locations across the southeastern United States and encompassed sand, loamy sand, and sandy loam soils across xeric and mesic moisture regimes (Fig. 1; Supplementary Table S3 & S4). Xeric and mesic groups formed distinct moisture classes, generating heterogeneous moisture-texture combinations across sites. Soil physicochemical properties varied across both texture and moisture gradients, with distinct nutrient patterns emerging within StressTexture combinations (Supplementary Table S4). Xeric sand soils were consistently low in organic matter (0.85-2.01%) and exhibited reduced overall nutrient availability, including lower total Nitrogen and NO_3_N concentrations. In contrast, mesic sand soils showed higher total nitrogen availability and NO_3_N values, along with slightly higher organic matter (0.84-2.27%). In loamy sand soils, mesic sites generally showed higher nutrient levels, particularly nitrogen pools, relative to xeric loamy sand soils, which exhibited comparatively lower values. Sandy loam soils displayed the greatest variability in physicochemical properties, with xeric sites including both low and high nutrient conditions (e.g., sites with elevated total nitrogen and NO_3_N), whereas mesic sandy loam soils showed more consistent nitrogen and micronutrient levels, including Zn. Soil pH remained acidic across all textures (3.8-6.4).

### 3.2 Alpha diversity responses to moisture stress are limited and texture-dependent

**Figure 2.**
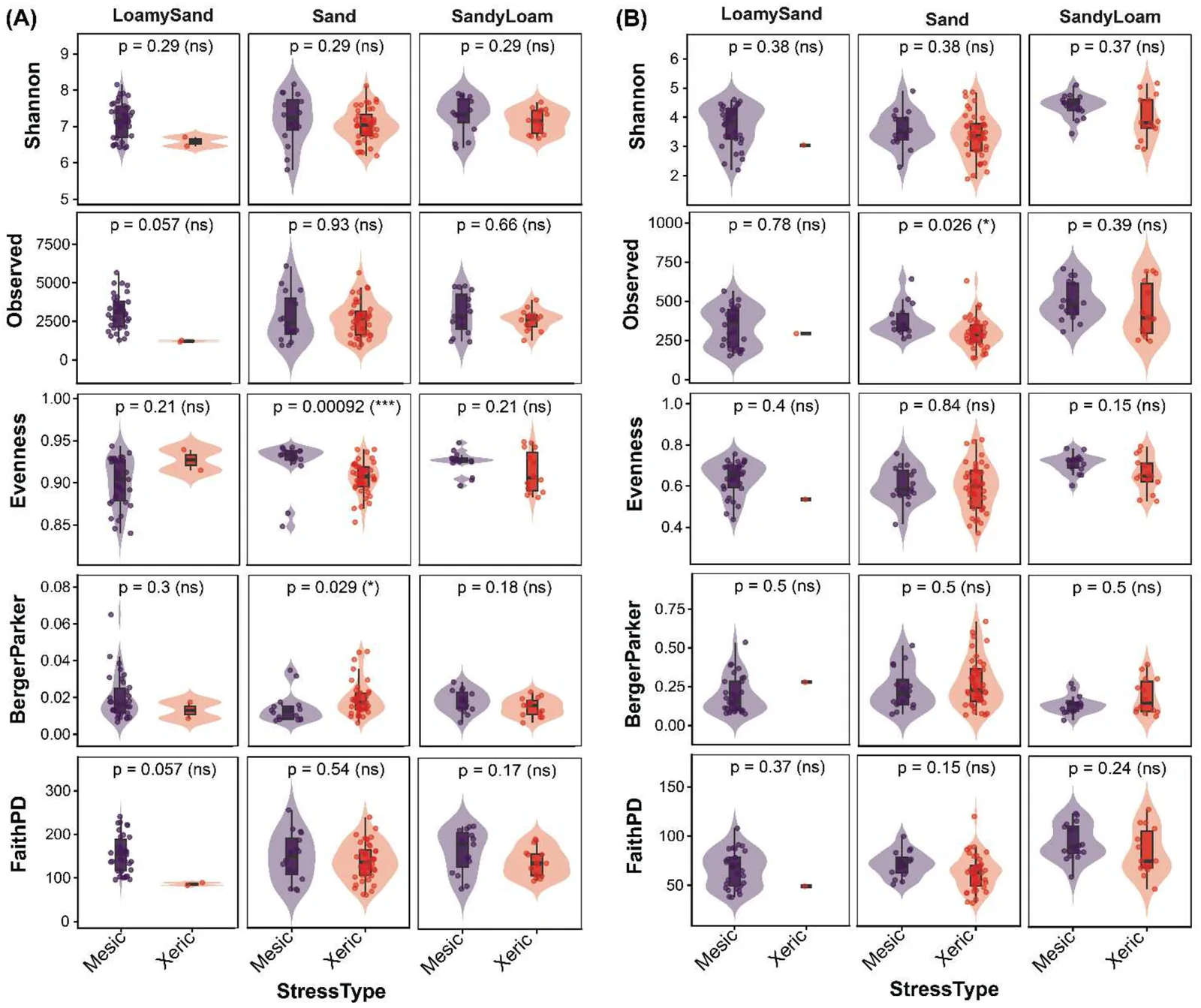
Bacterial and fungal α-diversity across soil textures and stress types. **(A)** Bacterial community α-diversity metrics. **(B)** Fungal community α-diversity metrics. Each panel displays violin plots with jittered points overlaid and boxplots for three soil textures (LoamySand, Sand, and SandyLoam) comparing Mesic and Xeric stress types. Violin shapes show distribution density; points represent individual samples; and boxplots indicate medians and interquartile ranges. P-value labels represent FDR-corrected significance and follow the significance scheme used in the analysis (* = p ≤ 0.05; ** = p ≤ 0.01; *** = p ≤ 0.001; ns = p > 0.05).

Bacterial and fungal α-diversity exhibited limited and texture-dependent responses to moisture stress (Fig. 2A–B). For bacteria, Shannon diversity, Observed richness, Berger-Parker dominance, and Faith’s PD showed no significant differences between mesic and xeric soils within any texture (Kruskal-Wallis, FDR-adjusted q > 0.29; Fig. 2A, top; Supplementary Table S5). In contrast, bacterial Pielou’s evenness index exhibited a significant shift only in sand, where xeric soils showed higher evenness compared to mesic soils (q < 0.001), indicating localized responses to moisture under low water-holding conditions, while loamy sand and sandy loam remained unchanged (q > 0.20; Fig. 2A, bottom).

Fungal α-diversity showed similarly limited responses to moisture stress. Shannon diversity, Peilou’s evenness, Berger-Parker dominance, and Faith’s PD showed no significant differences between mesic and xeric soils across all textures (FDR-adjusted q > 0.12, Fig. 2B, top). In contrast, Fungal Observed richness differed significantly between mesic and xeric conditions only within Sand soils, whereas no significant moisture-stress effects were detected within loamy sand or sandy loam (Fig. 2B, bottom; Supplementary Table S5).

### 3.3 Community β-Diversity differs with StressType and soil texture

**Figure 3.**
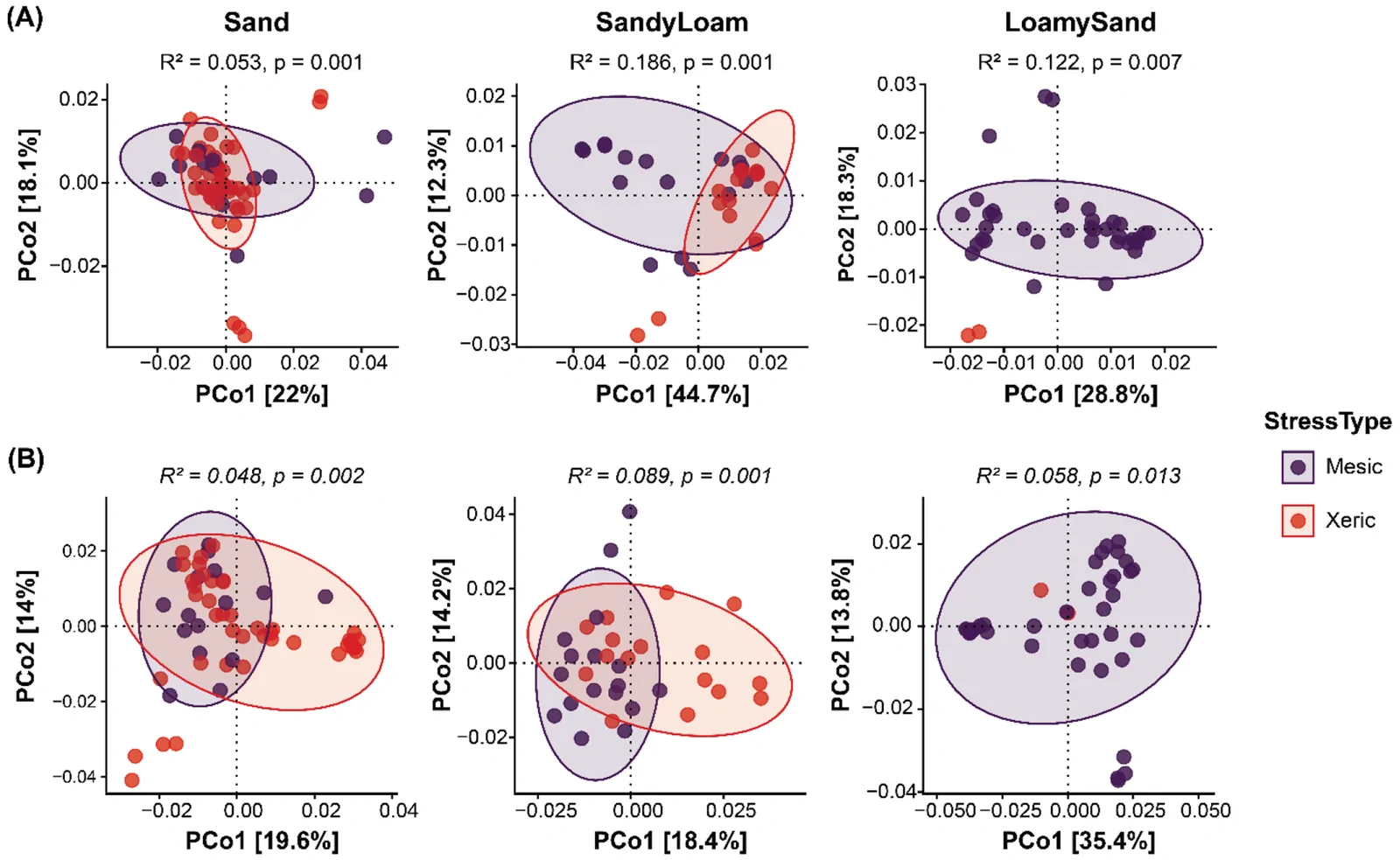
Bacterial and fungal β-diversity across soil textures and stress types. **(A)** Bacterial community β-diversity. **(B)** Fungal community β-diversity. Each panel displays PCoA ordinations derived from weighted UniFrac distance matrices for three soil textures (Sand, SandyLoam, and LoamySand), comparing mesic and xeric stress types. Points represent individual samples, and ellipses indicate 95% confidence regions for each stress group. Axis labels show the proportion of variation explained by PCoA1 and PCoA2. R^2^ and p-values correspond to PERMANOVA results.

Weighted UniFrac ordinations showed that bacterial community composition differed significantly across StressType, Texture, and StressTexture groupings, with the strongest separation observed across StressTexture combinations (PERMANOVA: R^2^ = 0.20, p = 0.001; Fig. 3A; Supplementary Fig. 1A–C). Texture explained greater variation than StressType alone (Texture: R^2^ = 0.09; StressType: R^2^ = 0.07; both p = 0.001). Within textures, mesic-xeric separation was strongest in sandy loam (R^2^ = 0.186, p = 0.001), followed by loamy sand (R^2^ = 0.122, p = 0.003) and sand (R^2^ = 0.053, p = 0.004). In contrast, bulk and rhizosphere communities showed minimal separation within textures (all R^2^ < 0.04; Supplementary Fig. 1G).

Fungal community composition also differed significantly across StressType, Texture, and StressTexture groupings (Fig. 3B; Supplementary Fig. 1D–F), although overall separation was weaker than in bacterial communities. StressTexture explained the greatest fungal variation (R^2^ = 0.152, p = 0.001), followed by Texture (R^2^ = 0.098, p = 0.001) and StressType (R^2^ = 0.045, p = 0.001). Within textures, mesic-xeric separation was again strongest in sandy loam (R^2^ = 0.089, p = 0.001), followed by loamy sand (R^2^ = 0.058, p = 0.010) and sand (R^2^ = 0.048, p = 0.003). As observed for bacterial communities, fungal bulk and rhizosphere communities showed minimal within-texture differentiation (Supplementary Fig. 1H). Collectively, these patterns indicate that interacting moisture-texture gradients exert stronger effects on microbial community composition than compartment differences, with bacterial communities responding more strongly than fungal communities.

### 3.4 Dominant bacterial and fungal taxa vary with soil texture

Bacterial communities were consistently dominated by *Alphaproteobacteria*, *Acidobacteriae*, *Actinobacteria*, and *Gammaproteobacteria* across all samples (Supplementary Fig. 2A). While these major classes were present in all textures, their relative proportions shifted primarily with soil texture.

*Acidobacteria and Verrucomicrobia* were visually more prominent in loamy sand compared to sand and sandy loam, whereas *Actinobacteria* and *Gammaproteobacteria* contributed a larger share of the community in sand and sandy loam. *Alphaproteobacteria* remained consistently abundant across textures but appeared proportionally higher in sandy loam than in loamy sand. In contrast, *Planctomycetes* showed relatively stable representation across all textures, without notable texture-driven variation. Within each texture, mesic and xeric soils exhibited broadly similar class-level profiles, indicating that moisture had limited influence on bacterial taxonomic composition relative to soil texture.

Fungal communities were dominated by *Agaricomycetes*, *Eurotiomycetes*, *Sordariomycetes*, and *Dothideomycetes*. (Supplementary Fig. 2B). Loamy sand was dominated by *Agaricomycetes*, whereas sand showed increased contributions from *Eurotiomycetes* and *Sordariomycetes*. Sandy loam exhibited a more even distribution of dominant classes, with notable contributions from *Dothideomycetes* and *Leotiomycetes*. These patterns indicate that fungal community composition is more strongly structured by soil texture than by soil moisture, consistent with texture-dependent variation in community composition observed in β-diversity analyses.

### 3.5 Environmental filtering of bacterial and fungal community composition

**Figure 4.**
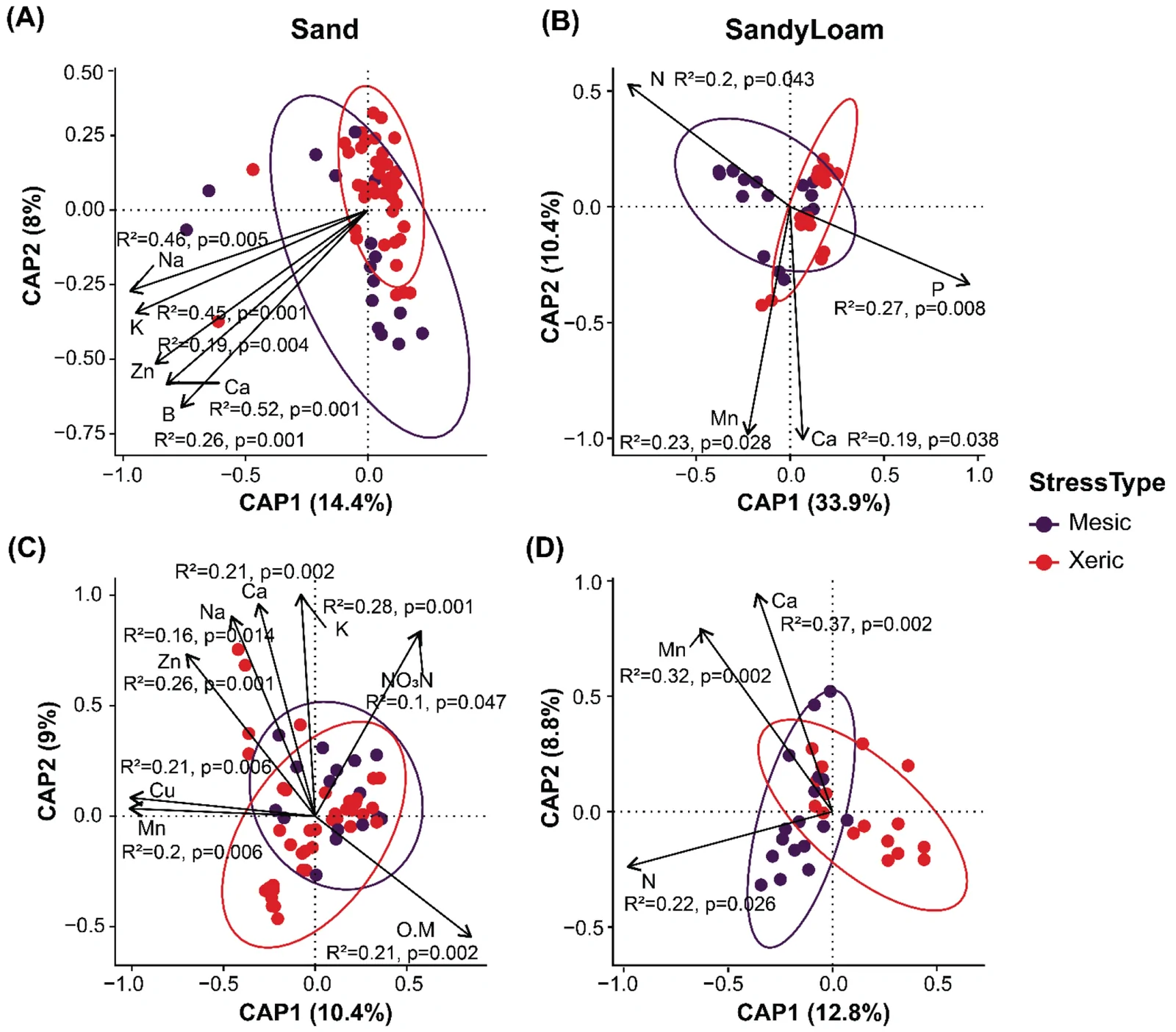
Distance-based redundancy analysis (dbRDA) of bacterial and fungal community composition across soil textures and stress regimes. **(A-B)** Bacterial dbRDA ordination in Sand and SandyLoam soils. **(C-D)** Fungal dbRDA ordination in Sand soils and SandyLoam soils. dbRDA panels display microbial community variation constrained by measured environmental variables, with points representing individual samples grouped by StressType. Arrows indicate environmental vectors, and ellipses represent 95% confidence regions for each StressType group. Axis labels show the proportion of constrained variation explained by dbRDA1 and dbRDA2. Corresponding R^2^ and p-values are shown for environmental variables included in the analysis.

dbRDA based on weighted UniFrac distances showed that bacterial community composition was significantly structured by StressType, Texture, and StressTexture groupings (all p ≤ 0.007; Supplementary Fig. 6A–C). Across StressType, bacterial community variation was most strongly associated with sodium (R^2^ = 0.20), nitrogen (R^2^ = 0.15), and soil pH (R^2^ = 0.11) (all p ≤ 0.002; Supplementary Fig. 6A). Across Texture and StressTexture groupings, nitrogen, soil pH, and organic matter remained the strongest drivers of bacterial community composition (R^2^ = 0.09-0.16, p ≤ 0.004; Supplementary Fig. 6B–C). Within sandy soils, bacterial communities were associated primarily with higher calcium, potassium, and sodium under mesic conditions (R^2^ = 0.19-0.52, p ≤ 0.004; Fig. 4A). In sandy loam, bacterial community variation was associated with phosphorus under xeric conditions and manganese and total nitrogen under mesic conditions (Fig. 4B).

Fungal community composition was also significantly structured by StressType, Texture, and StressTexture (all p = 0.001; Supplementary Fig. 6D–F). Across all grouping levels, the strongest drivers of fungal community variation were consistently nitrogen (R^2^ = 0.25), nitrite-N (R^2^ = 0.13-0.20), and magnesium (R^2^ = 0.11-0.13) (all p ≤ 0.004; Supplementary Fig. 6D–F). Within sandy soils, fungal communities were associated primarily with higher potassium, zinc, and organic matter under mesic conditions (R^2^ = 0.21-0.28, p ≤ 0.005; Fig. 4C), whereas sandy loam communities were associated with higher calcium, manganese, and total nitrogen under mesic conditions (Fig. 4D).

### 3.6 Community assembly processes shift across moisture and texture gradients

**Figure 5.**
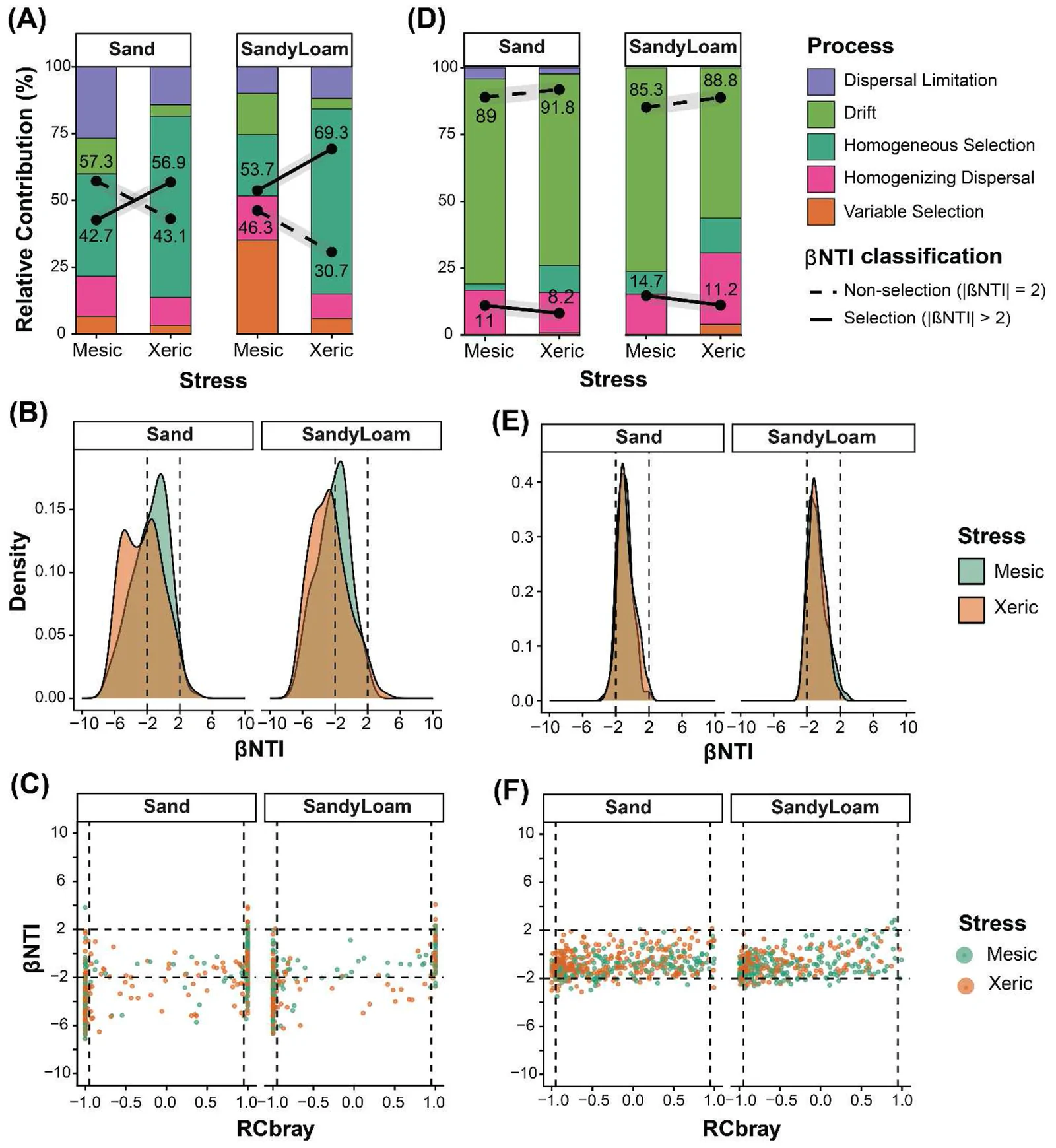
Community assembly patterns of bacterial and fungal communities across soil textures and stress types inferred from βNTI and RCbray analyses. Relative contributions of bacterial **(A)** and fungal **(D)** assembly processes across StressType groups within Sand and SandyLoam soils. **(B & E)** Density distributions of bacterial **(B)** and fungal **(E)** βNTI values across soil textures and stress types. Pairwise relationships between bacterial **(C)** and fungal **(F)** βNTI and RCbray values. Stacked bar plots show the relative contribution (%) of variable selection, homogeneous selection, dispersal limitation, homogenizing dispersal, and drift. Density plots display βNTI distributions with dashed vertical lines indicating null expectation thresholds (βNTI = ±2). Scatter plots show βNTI and RCbray relationships, with dashed lines indicating thresholds used for assembly process classification. Colors denote StressType groups.

Assembly processes differed between bacterial and fungal communities but responded consistently to moisture and soil texture conditions (Fig. 5A, D). Bacterial communities were dominated by homogeneous selection, which increased under xeric conditions (42.8% to 67.4%), accompanied by declines in drift (11.2% to 4.4%) and variable selection (17.6% to 4.3%). Mesic soils showed comparatively greater contributions from dispersal limitation and homogenizing dispersal. In sand soils, bacterial communities shifted from dispersal limitation under mesic conditions (21.0%) toward homogeneous selection under xeric conditions, whereas sandy loam communities showed elevated variable selection under mesic conditions (29.6%) but converged toward homogeneous selection under xeric conditions. In contrast, fungal communities remained dominated by drift across textures and moisture regimes (~82% mesic; ~74% xeric), with only modest increases in homogeneous selection and homogenizing dispersal under xeric conditions (Fig. 5D).

βNTI distributions supported these patterns (Fig. 5B, E). Bacterial communities shifted toward βNTI < −2 under xeric conditions across textures, consistent with stronger homogeneous selection, whereas mesic communities spanned broader stochastic and deterministic ranges. Fungal βNTI values remained largely within −2 to +2 regardless of texture, indicating predominantly stochastic assembly. Joint βNTI-RCbray space further resolved these patterns (Fig. 5C, F). Xeric bacterial communities clustered within the homogeneous selection region, whereas mesic communities extended into dispersal limitation and drift regions. In contrast, fungal communities remained centered within the drift-dominated region across textures, with only limited expansion into homogenizing dispersal and homogeneous selection under xeric conditions. Consistent with these patterns, the proportion of pairwise comparisons exceeding |βNTI| > 2 increased substantially in bacteria but only modestly in fungi (Fig. 5B, E). These results indicate stronger deterministic filtering in bacterial communities under xeric conditions, whereas fungal communities remained comparatively stochastic across moisture-texture gradients.

### 3.7 Bacterial and fungal co-occurrence networks differ across StressTexture conditions

**Figure 6.**
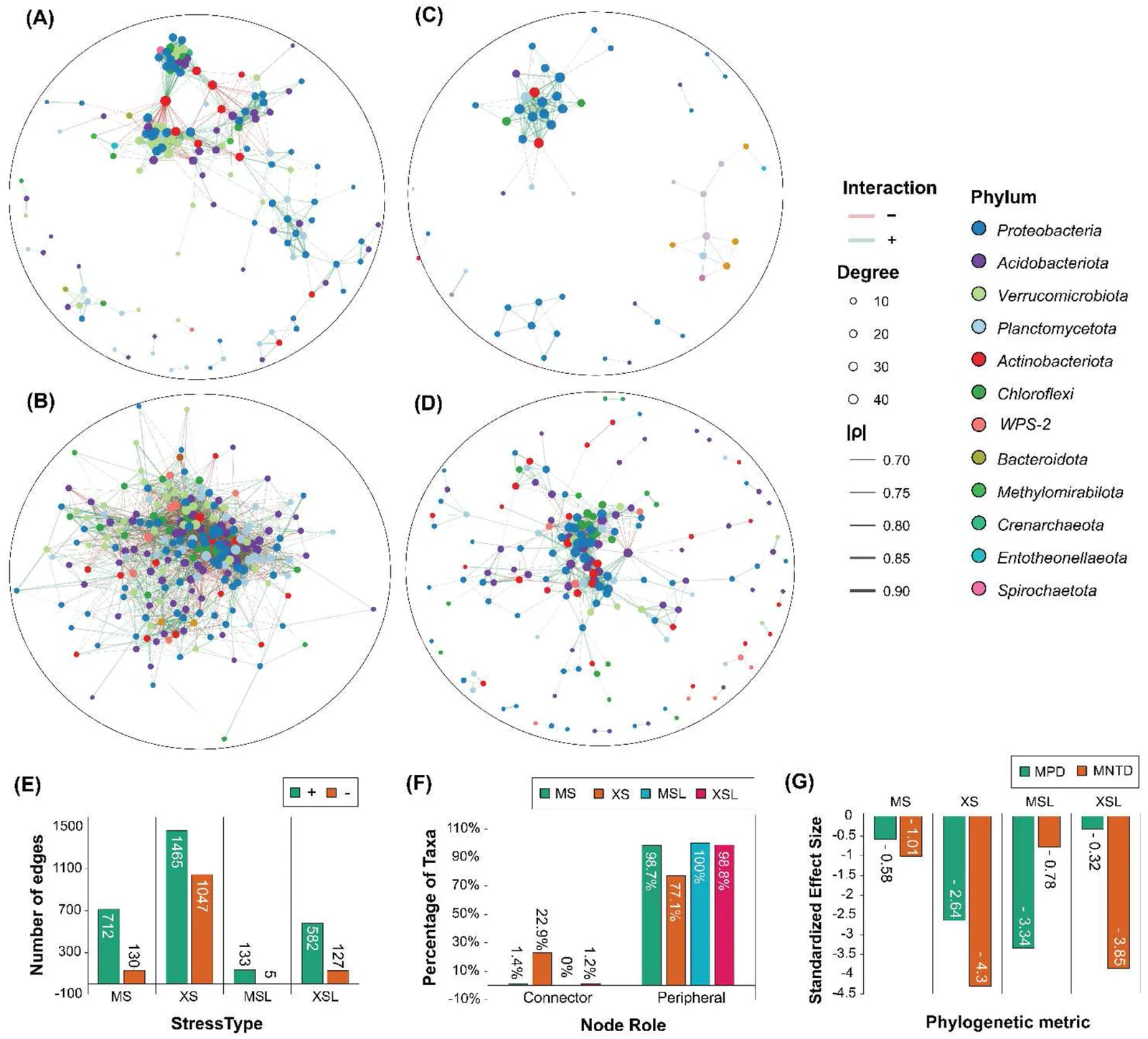
Co-occurrence network structure of bacterial communities across soil textures. **(A)** Mesic sand network. **(B)** Xeric sand network. **(C)** Mesic sandy loam network. **(D)** Xeric sandy loam network. Co-occurrence network constructed from 16S rRNA gene datasets, where nodes represent taxa and edges represent significant pairwise associations. Node size indicates degree (number of connections), node color corresponds to phylum-level taxonomy, and node fill reflects module membership (cluster structure). Edge colors denote positive or negative associations as indicated in the legend. Edge color indicates association type, where “+” represents positive correlations (co-occurrence) and “−” represents negative correlations (mutual exclusion). **(E)** Total number of positive (“+”) and negative (“−”) edges detected in each network. Positive edges indicate taxa with correlated occurrence patterns, whereas negative edges indicate inverse associations between taxa. **(F)** Distribution of node topological roles within each network. Abbreviations are as follows: MS, Mesic Sand; XS, Xeric Sand; MSL, Mesic Sandy Loam; XSL, Xeric Sandy Loam. Node roles were assigned based on within-module and among-module connectivity. **(G)** Standardized effect sizes (SES) of phylogenetic structure across treatments. Positive SES values indicate phylogenetic clustering, whereas negative SES values indicate phylogenetic overdispersion relative to null expectations.

Bacterial co-occurrence network structure varied strongly across StressTexture combinations, indicating that interacting moisture-texture gradients strongly influence microbial network organization (Fig. 6A–G). Xeric sand communities formed the largest and most connected networks (227 nodes, 2512 edges), whereas mesic sandy loam communities showed comparatively reduced network size and connectivity (54 nodes, 138 edges). Mesic sand (145 nodes, 842 edges) and xeric sandy loam (168 nodes, 709 edges) networks showed intermediate complexity. Across both textures, xeric networks contained higher proportions of negative interactions (sand: 41.7%; sandy loam: 17.9%) than mesic networks (sand: 15.4%; sandy loam: 3.7%), particularly in sandy soils, suggesting increased competitive or antagonistic interactions under moisture limitation (Fig. 6E). Xeric sand networks also contained a greater proportion of connector taxa (22.9%), whereas mesic sand and both sandy loam networks were dominated by peripheral taxa with few or no connectors (Fig. 6F). Despite differences in network architecture, all bacterial networks exhibited modular organization greater than null expectations (p < 0.001) (Fig. 6A–D). Phylogenetic structure further indicated stronger clustering under xeric conditions, particularly in xeric sand (SES_MPD = −2.64; SES_MNTD = −4.30) and xeric sandy loam (SES_MNTD = −3.85), consistent with intensified environmental filtering and deterministic assembly, whereas weaker clustering in mesic sand (SES_MPD = −0.58; SES_MNTD = −1.01) suggested reduced deterministic constraint (Fig. 6G).

**Figure 7.**
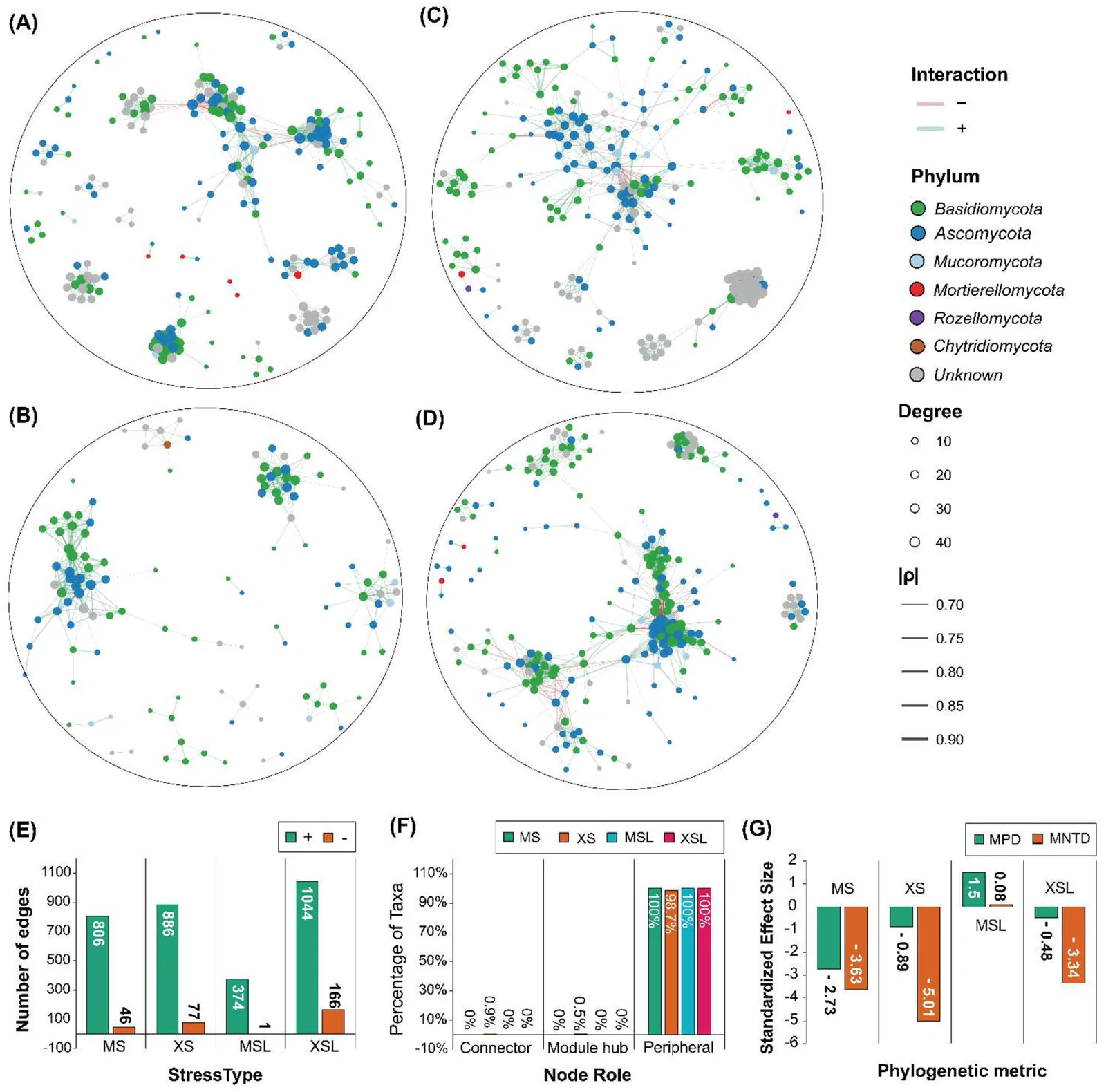
Co-occurrence network structure of fungal communities across soil textures. **(A)** Mesic sand network. **(B)** Xeric sand network. **(C)** Mesic sandy loam network. **(D)** Xeric sandy loam network. Co-occurrence network were constructed from ITS datasets, where nodes represent taxa and edges represent significant pairwise associations. Node size indicates degree (number of connections), node color corresponds to phylum-level taxonomy, and node fill reflects module membership (cluster structure). Edge color indicates association type, where “+” represents positive correlations (co-occurrence) and “−” represents negative correlations (mutual exclusion). **(E)** Total number of positive (“+”) and negative (“−”) edges detected in each network. Positive edges indicate taxa with correlated occurrence patterns, whereas negative edges indicate inverse associations between taxa. **(F)** Distribution of node topological roles within each network. Abbreviations are as follows: MS, Mesic Sand; XS, Xeric Sand; MSL, Mesic Sandy Loam; XSL, Xeric Sandy Loam. Node roles were assigned based on within-module and among-module connectivity. **(G)** Standardized effect sizes (SES) of phylogenetic structure across treatments. Positive SES values indicate phylogenetic clustering, whereas negative SES values indicate phylogenetic overdispersion relative to null expectations.

Fungal co-occurrence network structure also varied across StressTexture combinations, although the patterns differed substantially from those of bacterial networks (Fig. 7A–G). Xeric sandy loam communities formed the largest and most connected fungal networks (108 nodes, 1210 edges), whereas mesic sandy loam communities showed comparatively reduced connectivity (60 nodes, 375 edges). Xeric sand (117 nodes, 963 edges) and mesic sand (91 nodes, 852 edges) networks showed intermediate complexity. Across all StressTexture groups, fungal networks were strongly dominated by positive associations (86.3-99.7%), with relatively few negative interactions compared to bacterial networks (Fig. 7E). Node role distributions were highly simplified, with peripheral taxa comprising >98% of nodes across all treatments and only xeric sand containing connectors (0.9%) and a single module hub (0.45%) (Fig. 7F). Despite differences in connectivity, all fungal networks exhibited modular organization greater than null expectations (Q = 0.67-0.82; p < 0.001) (Fig. 7A–D). Phylogenetic structure varied across StressTexture groups, with xeric sand (SES_MPD = −0.89; SES_MNTD = −5.01), mesic sand (SES_MPD = −2.73; SES_MNTD = −3.63), and xeric sandy loam (SES_MNTD = −3.34) showing phylogenetic clustering consistent with environmental filtering, whereas mesic sandy loam exhibited weaker phylogenetic constraint and greater overdispersion (SES_MPD = 1.50; Fig. 7G).

### 3.8 Predicted microbial functions shift with StressType within soil texture

**Figure 8.**
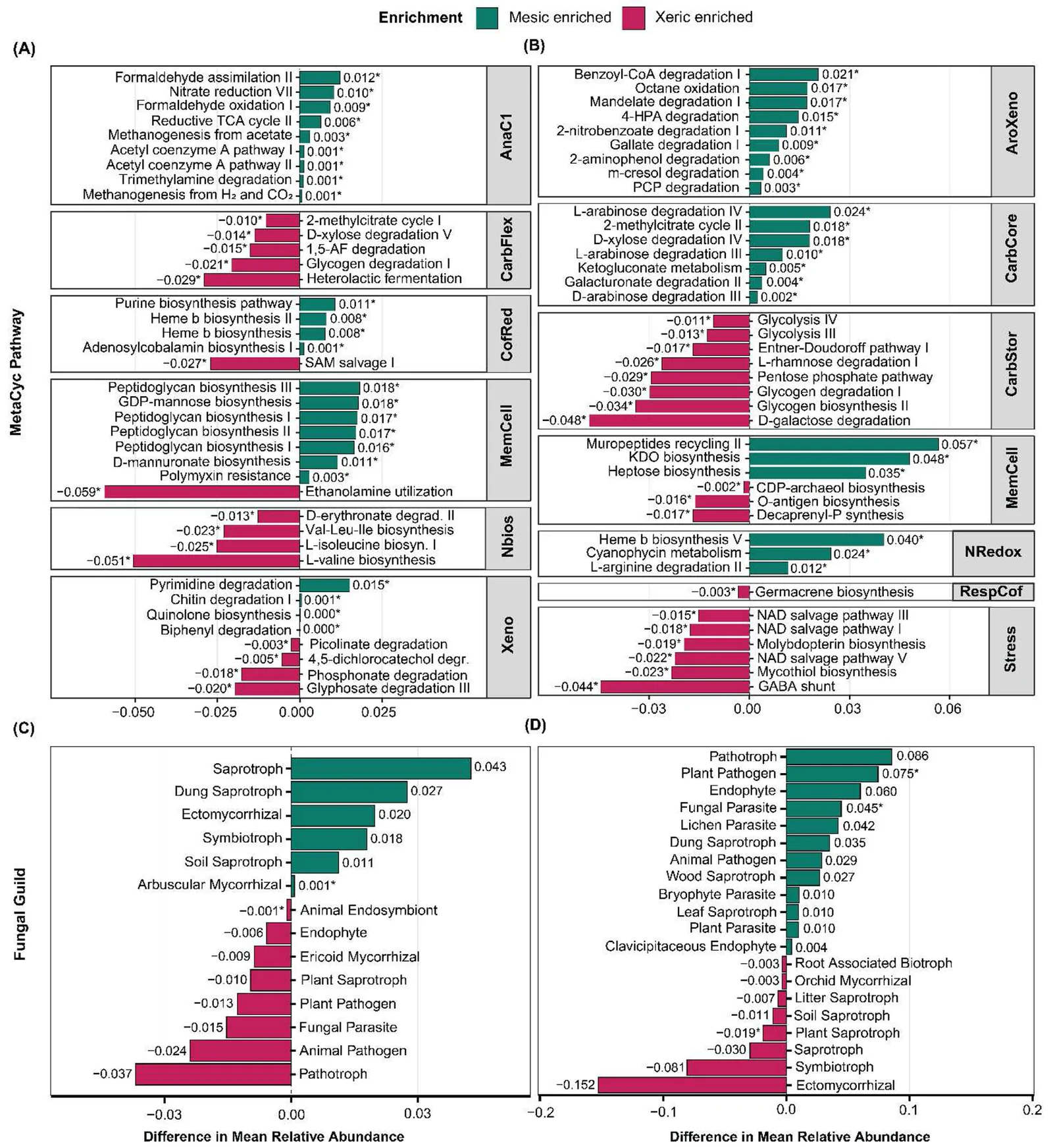
Predicted functional profiles of bacterial and fungal communities across soil textures. **(A-B)** MetaCyc pathway shifts in Sand and SandyLoam soils. Only pathways that differ significantly between mesic and xeric conditions are shown (adjusted *P* < 0.05). Pathways are grouped according to functional category. Functional pathway categories include: *Anaerobic & C1 Metabolism (AnaC1); Aromatic & Xenobiotic Turnover (AroXeno); Carbon Reorganization, Storage & Fermentation (CarbFlex); Core Carbon Metabolism & Stress Detox (CarbCore); Carbon Storage & Energy Metabolism (CarbStor); Cofactor & Redox Biosynthesis (CofRed); Membrane & Cell Wall Investment (MemCell); Nitrogen & Amino Acid Biosynthesis (Nbios); Nitrogen Cycling, Redox & Cofactors (NRedox); Respiratory Cofactors & Quinones (RespCof); Stress Defense & Osmoprotection (Stress); and Xenobiotic & Competitive Carbon Use (Xeno)*. **(C-D)** Fungal guild shifts in Sand and SandyLoam soils. Guilds are classified according to ecological function. Asterisks (*) indicate significant differences between treatments (adjusted *P* < 0.05). Bars represent differences in mean relative abundance, with positive values indicating mesic enrichment and negative values indicating xeric enrichment.

Predicted functional profiles differed between mesic and xeric conditions in a texture-dependent manner (Fig. 8A–B). In sand soils, mesic communities were enriched in C1 and redox-associated pathways, including formaldehyde assimilation, nitrate reduction, and methanogenesis, whereas xeric communities showed higher relative abundance of carbohydrate degradation, including the 2-methylcitrate cycle, *D*-xylose degradation, glycogen degradation, and heterolactic fermentation (Fig. 8A), indicating enrichment of substrate degradation and stress-associated metabolism under low moisture conditions. In sandy loam soils, mesic communities were enriched in pathways associated with aromatic compound degradation and complex carbon turnover, whereas xeric communities showed greater representation of central carbon metabolism and energy-conservation pathways, including glycolysis, pentose phosphate metabolism, and NAD salvage pathways (Fig. 8B).

Fungal functional composition also varied across StressTexture groups (Fig. 8C–E). Functional profiles were dominated by symbiotrophic and ectomycorrhizal guilds, followed by saprotrophs (Fig. 8C). In sandy soils, mesic conditions were associated with higher relative abundance of saprotrophic guilds, whereas xeric conditions showed greater representation of pathotrophic guilds (Fig. 8D). In sandy loam soils, mesic communities exhibited greater abundance of multiple symbiotic and decomposer-associated guilds, whereas xeric conditions were associated with declines in ectomycorrhizal and plant saprotrophic fungi and increased representation of stress-associated guilds (Fig. 8E). Overall, these patterns indicate a shift from symbiotic and decomposer strategies under mesic conditions toward stress-associated and pathotrophic strategies under xeric conditions.

## 4 DISCUSSION

### 4.1 Soil texture modulates microbial responses to moisture limitation

Although drought is often framed as a dominant ecological filter in soils [5], our results show that its effects vary across soil textures. In this study, moisture did not uniformly structure *Andropogon virginicus* rhizosphere communities across sites; instead, its effects were mediated by soil texture, which controlled microscale water retention, diffusion, and availability. The strongest mesic-xeric separation in community composition occurred in sandy soils, where rapid drainage and limited water retention likely limited substrate diffusion and reduced microbial access to nutrients. Under these conditions, water, carbon and other nutrients limitations likely narrow viable niches, favoring drought-tolerant taxa. In contrast, loam soils with better water retention and higher pore-water connectivity during drying reduce the strength of moisture-driven environmental filtering on microbial communities. This pattern aligns with a mechanistic view of soil as a spatially structured habitat, where texture determines pore size distribution, connectivity, and water retention, which in turn regulate oxygen diffusion, substrate accessibility, and microbial dispersal [66, 67]. In sandy soils, discontinuities in water channels limit the diffusion of dissolved substrates, create isolation among microbes, and increase resource competition within fragmented habitats, whereas finer-textured soils better maintain continuous water films to support microbial activity under low moisture conditions [68, 69]. Experimental studies on drought also showed stronger microbial stress responses in sandy soils compared to loams or clays [70, 71]. Similar context-dependent outcomes have also been reported in *Andropogon gerardii*, where soil matrix properties influence plant–microbe interactions [72].

However, texture-dependent modulation of drought effects does not apply equally to all systems. Moisture can override textural limitations in a system, depending on the ecosystem context, such as one rich in organic matter or dominated by plants [5, 70, 73]. In *Andropogon gerardii*, microbial communities exhibited strong local adaptation and differed between native and non-native soil environments in their ability to enhance plant performance, indicating that environmental history and site-specific conditions can override generalizable patterns [74]. Similarly, legacy effects and prior exposure to drying-rewetting cycles can alter microbial tolerance to drought, weakening texture dependence [75–77]. The consistent StressTexture-driven patterns in this study suggest that soil texture defines the ecological context within which drought operates. Moisture provides the immediate selective pressure; soil texture regulates how that pressure is experienced within the microbial habitat. This interaction provides a mechanistic explanation for variability in drought responses across ecosystems and highlights the need to consider soil physical parameters alongside other properties.

In addition to soil texture and moisture, nutrient availability acts as a co-driver of microbial assembly. Dominant environmental variables structuring community variation differed among soil textures, indicating context-dependent environmental filtering rather than a single controlling gradient. In sandy soils, bacterial and fungal communities were primarily associated with nutrient availability under mesic conditions, particularly exchangeable cations, micronutrients, and organic matter, consistent with increased nutrient diffusion and microbial activity under higher moisture availability. In sandy loam, bacterial communities were associated with phosphorus under xeric conditions, whereas fungal communities were associated with calcium, manganese, and total nitrogen under mesic conditions, indicating divergent bacterial and fungal responses across nutrient axes [75, 78]. Across StressType, Texture, and StressTexture groupings, bacterial communities were repeatedly associated with nitrogen, soil pH, and organic matter, whereas fungal communities were consistently associated with nitrogen-related variables, particularly NO3N and magnesium, supporting a recurring role of nutrient-mediated environmental filtering across moisture gradients [79–81]. Together, these findings suggest that soil texture modifies how moisture and nutrient gradients structure microbial communities.

### 4.2 Moisture limitation restructures community composition and interactions without reducing diversity

Xeric conditions had limited effects on within-sample diversity but instead altered community composition and interactions across StressTexture groups. Alpha diversity remained largely stable, particularly in bacterial communities, suggesting that richness and evenness were buffered despite stress. This pattern is widely reported across ecosystems, where microbial richness is maintained even under environmental stresses [82, 83]. Such buffering is often attributed to functional redundancy, in which multiple taxa contribute to similar ecological functions that sustain ecosystem processes despite compositional shifts [84, 85]. In contrast, fungal diversity showed greater sensitivity to moisture and texture, indicating taxon-specific responses across StressTexture groups, consistent with studies showing stronger environmental control over fungal diversity and distribution [86, 87]. The greater responsiveness of fungal α-diversity, however, suggests tighter ecological constraints and potentially lower redundancy under shifting environmental conditions. Together, this suggests that StressTexture filtering in this system primarily acts through species identity and interactions, rather than through the number of taxa present. Instead of reducing richness, communities were reorganized, as reflected by differential abundance patterns across StressTexture conditions and further supported by network analysis and predicted functional profiles. In a cross-inoculation study, rhizosphere microbiomes from *Andropogon virginicus* altered microbial community structure and network complexity in maize under drought conditions [11]. Evidence from grassland and agricultural systems similarly shows that drought primarily restructures microbial community composition and interaction networks, while effects on overall richness are often limited or inconsistent [70, 88, 89].

In contrast, beta-diversity analyses showed a clear mesic-xeric separation across soil textures, suggesting that ecological patterns are driven by compositional shifts within communities rather than by diversity loss. Similarly, bacterial assembly results showed stronger homogeneous selection and increased deterministic assembly under xeric conditions based on βNTI and RCbray analyses. These patterns are indicative of niche-based assembly, where environmental drivers select for taxa with distinct ecological and physiological traits [90]. Importantly, these shifts in composition were also reflected in altered taxon associations, indicating that changes in community structure include both taxa and their interactions [70]. The weak separation between rhizosphere and bulk soils indicates that StressTexture filtering exerts a stronger influence than plant-associated effects. This contrasts with findings in *Andropogon gerardii*, where host identity has been shown to strongly structure rhizobiome assembly under abiotic stress [91]. Many other studies report strong rhizosphere effects driven by root exudation and plant-microbe interactions [92, 93], which may be diminished in ecosystems characterized by continuous vegetation cover. In our study, dense stands of *Andropogon virginicus*, overlapping root systems likely extend rhizosphere influence beyond discrete zones, reducing spatial heterogeneity. Similar reductions in rhizosphere-bulk separation have been reported in grassland systems with high root density [94]. Drought may also reduce plant-mediated selection by limiting microbial survival through reduced moisture availability rather than through root inputs [73]. As a result, ecological change is reflected more in compositional shifts and interaction restructuring than in diversity alone.

### 4.3 Bacterial and fungal communities respond differently to moisture limitation

Bacterial and fungal communities responded differently to StressTexture filtering, reflecting distinct ecological strategies. Bacterial communities showed greater compositional shifts, stronger associations with environmental variables, and higher restructuring of interaction networks than fungal communities. Under xeric conditions, particularly in sandy soils, bacterial networks were larger and more connected, with a higher proportion of negative associations. This finding aligns with ecological theory, which predicts that resource limitation increases competition as niche space contracts [95]. Empirical studies of microbial networks support this interpretation, that environmental stress can increase interaction strength and alter network structure, often leading to a higher proportion of negative associations [96, 97], resulting in strong associations among taxa. The occurrence of connector taxa in xeric networks further suggests increased integration across modules, potentially maintaining network organization under stress [98]. Such taxa likely support connectivity within the community.

In contrast, fungal communities exhibited more conservative responses. Network structure was characterized by predominantly positive associations and high modularity, with few connector taxa, suggesting limited reorganization of interactions and greater dependence on stable niche structure. The filamentous growth of fungi allows them to span spatial heterogeneity and access resources across microsites [99]. This growth form may buffer fungi against localized resource limitation and reduce the need for rapid network restructuring. Fungal responses were reflected through changes in functional guild composition. In sand, xeric conditions favored pathotrophic and stress-associated guilds, whereas mesic conditions supported symbiotic and decomposer guilds, consistent with differences in resource availability. Similar drought-driven shifts in fungal guilds have been reported in forest and grassland ecosystems [87]. In sandy loam, enrichment of symbiotrophic and ectomycorrhizal guilds under xeric conditions may also relate to the ecological roles of common mycorrhizal networks which in *Andropogon gerardii*, have been shown to influence plant survival and plant-plant interactions under drought conditions [100].

Phylogenetic patterns further emphasize these differences. Strong clustering in bacterial communities under xeric conditions suggests deterministic filtering favoring closely related taxa with shared drought-adaptive traits [51]. In contrast, fungal communities exhibited weaker clustering or overdispersion under mesic conditions, indicating greater niche differentiation when environmental constraints are relaxed. These findings support a model in which bacteria respond rapidly to environmental change through compositional and interaction restructuring, whereas fungi maintain more compartmentalized and functionally specialized communities.

Bacterial and fungal communities also differed in assembly dynamics. Xeric conditions increased deterministic assembly in bacterial communities, particularly through homogeneous selection, whereas fungal communities remained dominated by stochastic processes across textures. Similar drought-associated increases in deterministic bacterial assembly have been linked to reduced pore-water connectivity and stronger environmental filtering under dry conditions [50, 51, 101] consistent with broader stochastic-deterministic community assembly frameworks [102]. In contrast, fungal communities remained largely stochastic across moisture regimes, consistent with reports of greater stochasticity and dispersal limitation in fungal community assembly [70, 87, 103]. Together, these patterns indicate that xeric conditions deterministic filtering primarily in bacterial communities, whereas fungal communities remain comparatively buffered against moisture-driven selection.

### 4.4 Network and functional shifts reveal integrated mechanisms of moisture-texture responses

The strongest ecological responses were observed in network structure and predicted functions, with moisture-texture groups driving coordinated shifts in interactions and metabolic strategies. In xeric sand networks, increased connectivity may enhance stability by maintaining pathways for resource flow and metabolic exchange. Network theory suggests that highly connected systems can buffer perturbations by distributing effects across multiple interactions [104]. The presence of connector taxa further supports the idea that certain organisms play key roles in network resilience under stress. In mesic soils, networks were less connected and dominated by positive associations, likely suggesting reduced stress filtering and broader niche overlap. However, positive correlations in co-occurrence networks often reflect shared environmental preferences rather than true cooperative interactions [105, 106].

Predicted functional profiles aligned with changes in network connectivity and interaction patterns. Xeric bacterial communities were enriched in pathways related to energy conservation, stress tolerance, and substrate degradation, reflecting adaptation to resource-limited environments [5, 107]. Increased representation of carbohydrate degradation and fermentation pathways further suggests a shift toward exploiting available substrates under stress, a pattern widely observed in drought-affected soils where microbes prioritize maintenance over growth [108, 109]. These functional patterns may also reflect underlying nutrient limitation, particularly nitrogen availability, which can influence microbial and plant-associated processes. In *Andropogon gerardii*, nitrogen availability has been shown to influence plant-fungus interactions, with reduced growth benefits under low nitrogen conditions [110], while rhizosphere-associated *Pseudomonas* isolates exhibited traits linked to drought tolerance and plant growth promotion [13]. Mesic communities were enriched in pathways associated with nutrient cycling and complex carbon utilization, suggesting higher metabolic activity and resource availability. These findings align with studies demonstrating increased microbial processing of carbon and nutrients under higher soil moisture [107, 111]. Fungal functional profiles showed similar trends, with xeric conditions favoring stress-associated and pathotrophic guilds and mesic conditions supporting symbiotic and decomposer strategies. Such shifts are consistent with studies showing that drought alters fungal functional composition and increases the prevalence of stress-tolerant or pathogenic taxa [87, 112]. These changes likely reflect shifts in plant health, substrate availability, and competitive interactions under drought, which can restructure plant-fungal associations [73, 113].

Notably, prior work has shown that microbial functions can remain stable despite taxonomic turnover, reflecting functional redundancy within communities [84, 85]. However, drought-driven shifts in soil microbial composition and interaction structure can also alter functional organization and metabolic prioritization [70, 114]. Here, coordinated changes in community composition, interaction networks and predicted functional profiles suggest that moisture-texture filtering reorganizes microbial functional priorities rather than causing broad functional loss.

## 5 Conclusions

Microbial assembly in *A. virginicus* soils is governed by interacting environmental filters, in which soil texture defines the physical framework that modulates how moisture stress is experienced at the microscale. Rather than reducing diversity, limited soil moisture primarily reorganized community composition, assembly processes, interaction networks, and functional potential across StressTexture gradients. These responses diverged between domains, reflecting contrasting ecological strategies. Bacterial communities exhibited stronger compositional shifts, increased deterministic assembly under xeric conditions and greater restructuring of interaction networks, whereas fungal communities remained comparatively stochastic, modular and functionally compartmentalized across moisture regimes. Coordinated shifts in predicted functional profiles alongside taxonomic and network reorganization further suggest that moisture-texture filtering reorganizes microbial functional priorities rather than eliminating functional capacity. This supports a context-dependent view of functional redundancy, in which ecosystem function is maintained through redistribution of metabolic emphasis rather than strict functional stability. Overall, these findings support a context-dependent view of microbial resilience, in which soil texture modifies how moisture and nutrient gradients structure microbiome organization and ecological function. Integrating compositional, assembly, network, and functional perspectives reveals that microbial responses to environmental stress in this system are inherently multidimensional and cannot be captured by diversity metrics alone. As xeric conditions intensify under climate change, the interaction between moisture availability and soil physical structure will likely play a central role in determining microbiome resilience and ecosystem functioning across heterogeneous landscapes.

## Supplementary Material

1

This is a list of supplementary files associated with this preprint. Click to download.

- AdditionalFile1SupplementaryTables.xlsx
- AdditionalFile2SupplementaryResults.docx

## Data Availability

Raw sequencing reads have been deposited in the NCBI Sequence Read Archive (SRA) under BioProject PRJNA1465459. BioSample accessions range from SAMN59746056 to SAMN59746187. Metadata describing sample locations, soil physicochemical properties, and environmental variables are provided in Additional_File_1_Supplementary_Tables and correspond directly to the deposited BioSample identifiers. All scripts used for sequence processing, statistical analyses, and figure generation are publicly available at GitHub (https://github.com/Muhammadshoibnawaz/microbiome-ecology-workflow)
