# Supplementary material for: Soil texture mediates microbial assembly and network organization under moisture stress
Source: Res Sq. 2026 Aug 3:rs.3.rs-10233190. Preprint. [Version 1] doi: 10.21203/rs.3.rs-10233190/v1 (PMC13484798; doi:10.21203/rs.3.rs-10233190/v1)
Supplement: Supplementary file 1 [file NIHPPrs10233190v1-supplement-1.pdf]

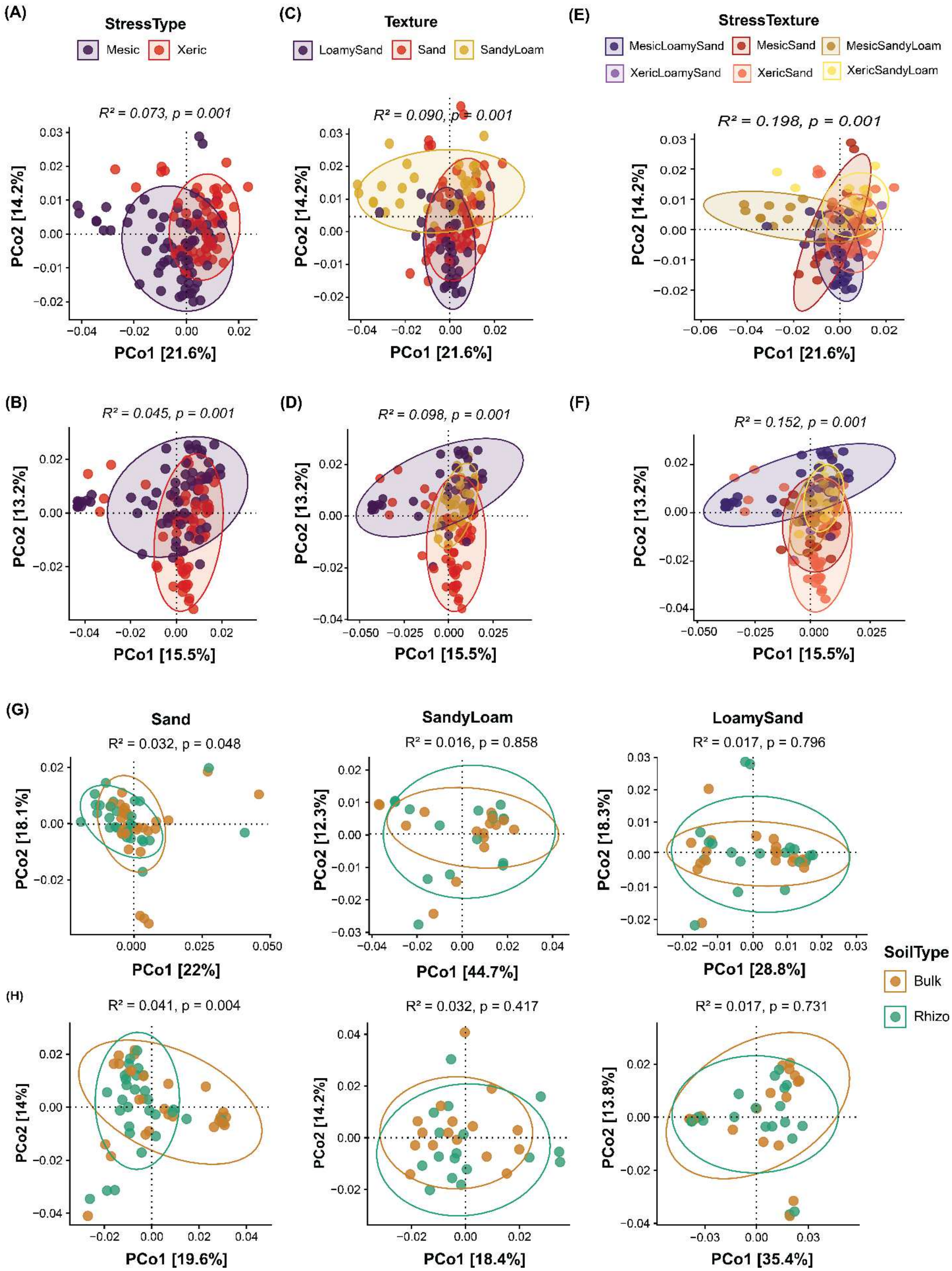
(A)
StressType
Mesic
Xeric
R² = 0.073, p = 0.001
PCo2 [14.2%]
PCo1 [21.6%]
(C)
Texture
LoamySand
Sand
SandyLoam
R² = 0.090, p = 0.001
PCo2 [14.2%]
PCo1 [21.6%]
(E)
StressTexture
MesicLoamySand
MesicSand
MesicSandyLoam
XericLoamySand
XericSand
XericSandyLoam
R² = 0.198, p = 0.001
PCo2 [14.2%]
PCo1 [21.6%]
(B)
R² = 0.045, p = 0.001
PCo2 [13.2%]
PCo1 [15.5%]
(D)
R² = 0.098, p = 0.001
PCo2 [13.2%]
PCo1 [15.5%]
(F)
R² = 0.152, p = 0.001
PCo2 [13.2%]
PCo1 [15.5%]
(G)
Sand
R² = 0.032, p = 0.048
PCo2 [18.1%]
PCo1 [22%]
SandyLoam
R² = 0.016, p = 0.858
PCo2 [12.3%]
PCo1 [44.7%]
LoamySand
R² = 0.017, p = 0.796
PCo2 [18.3%]
PCo1 [28.8%]
SoilType
Bulk
Rhizo
(H)
R² = 0.041, p = 0.004
PCo2 [14%]
PCo1 [19.6%]
R² = 0.032, p = 0.417
PCo2 [14.2%]
PCo1 [18.4%]
R² = 0.017, p = 0.731
PCo2 [13.8%]
PCo1 [35.4%]

348

Supplementary Figure 1. Bacterial and fungal β-diversity based on weighted UniFrac distances across StressType, Texture, StressTexture and SoilType.

Bacterial **(A, C, E, F)** and Fungal **(B, D, F, H)** community weighted UniFrac PCoA ordinations. **(A-B)** StressType (Mesic vs. Xeric). **(C-D)** Texture (LoamySand, Sand, SandyLoam). **(E-F)** Combined StressTexture classes. **(G-H)** SoilType (Bulk vs. Rhizo) within each Texture.

Each panel displays a PCoA plot derived from weighted UniFrac distances, with points representing individual samples and ellipses indicating 95% confidence regions for each group. Axis labels show the percentage of variation explained by PCoA1 and PCoA2. R^2 and p-values correspond to PERMANOVA results.

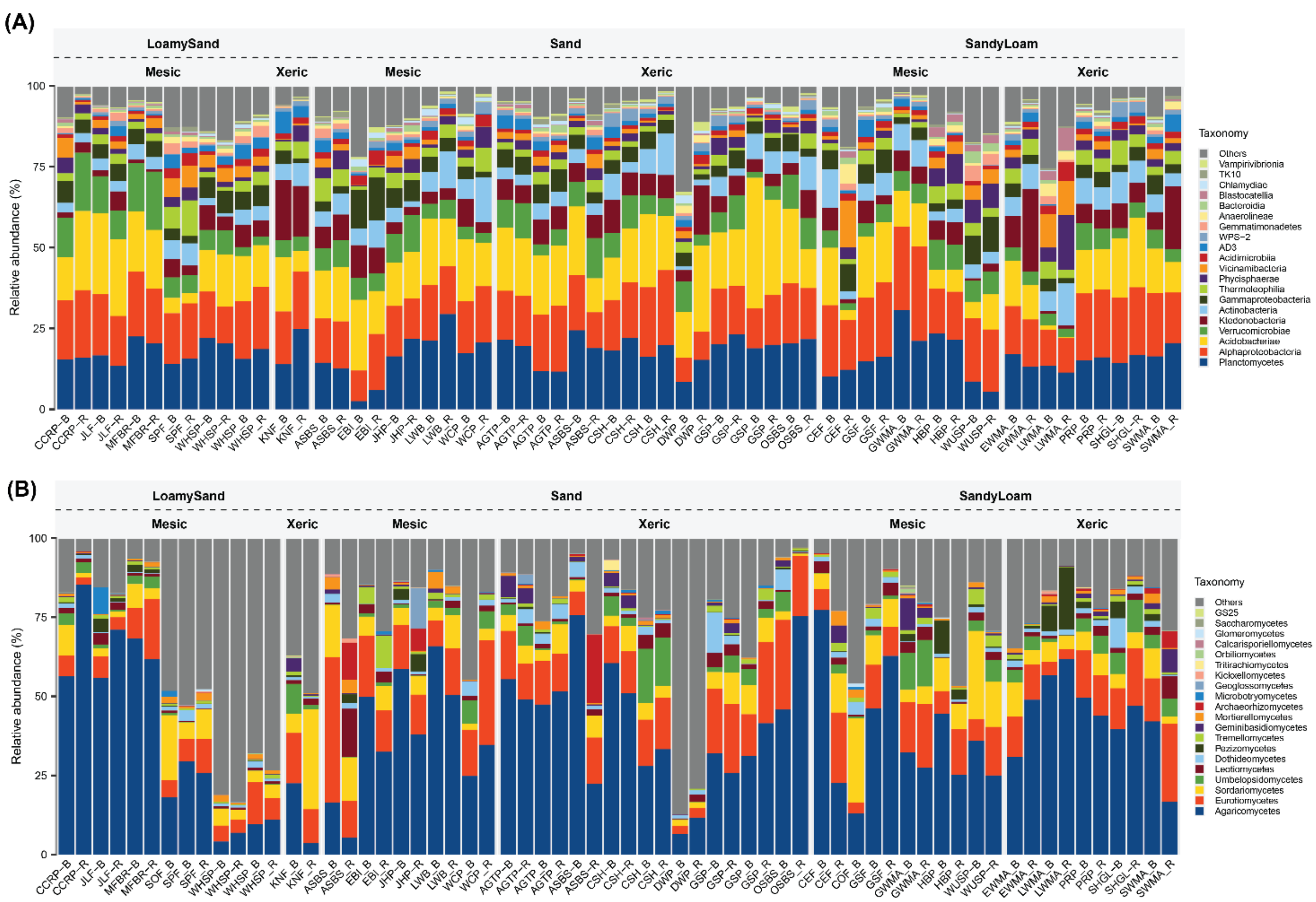

Supplementary Figure 2. Order-level bacterial and fungal community composition across soil textures and stress types.

(A) Bacterial community taxonomic composition. **(B)** Fungal community taxonomic composition. Each panel displays stacked bar plots for three soil textures (loamy sand, sand, and sandy loam),

comparing mesic (M) and xeric (X) stress types. Bars represent individual samples, and colors correspond to the relative abundance of dominant taxonomic classes as shown in the legend.

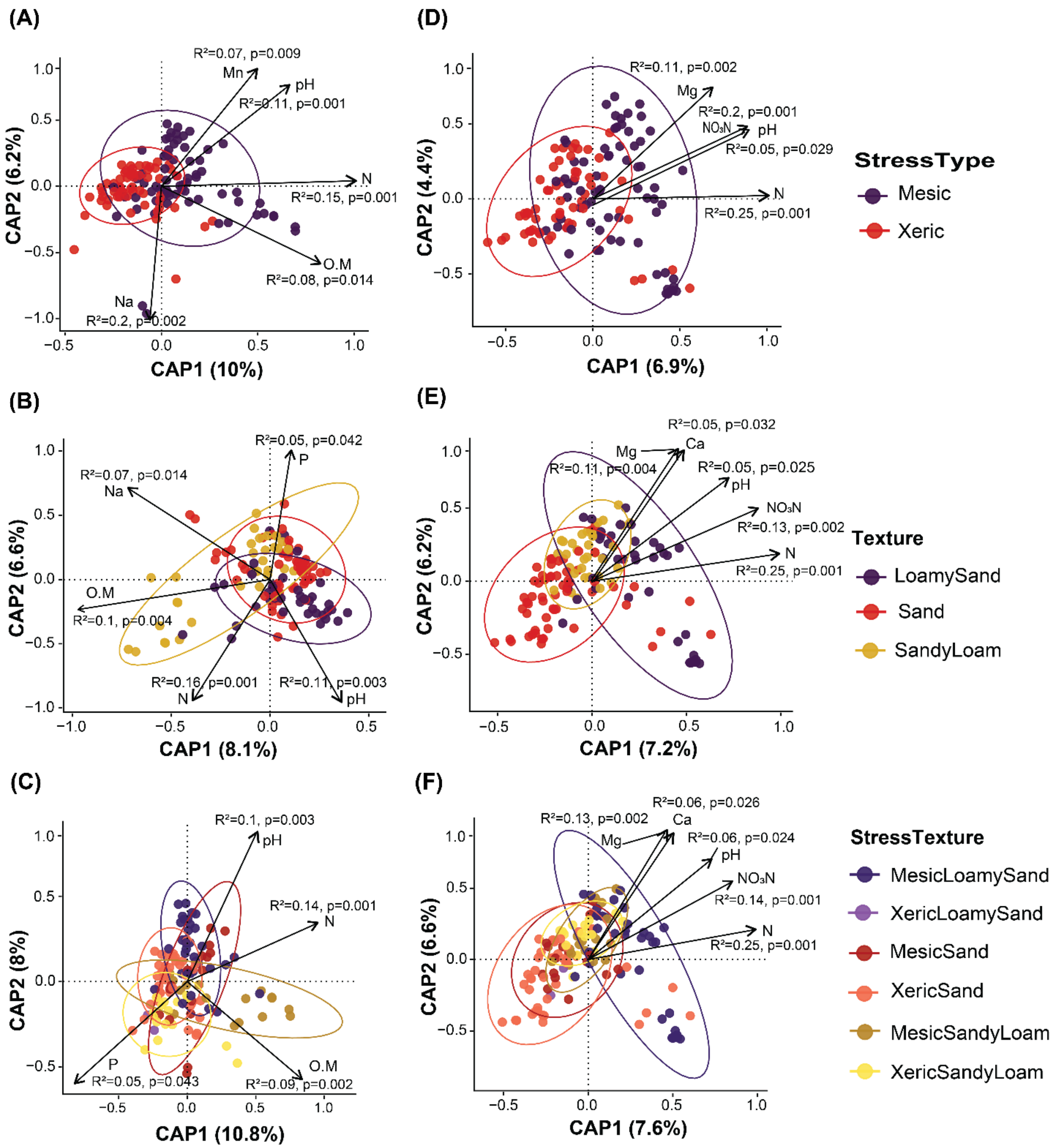

Supplementary Figure 6. Distance-based redundancy analysis (dbRDA) of bacterial and fungal community composition constrained by soil physicochemical variables.

Panels show bacterial **(A-C)** and fungal **(D-F)** communities grouped by StressType, Texture, and StressTexture, respectively. Points represent individual samples, and ellipses denote 95% confidence intervals for group centroids. Arrows indicate significant environmental variables fitted to ordination space, with associated R^2 and P values shown. Percent variation explained by each constrained axis is indicated on the axis labels.
